# Targeting TGF-**β** for treatment of osteogenesis imperfecta

**DOI:** 10.1172/JCI152571

**Published:** 2022-04-01

**Authors:** I-Wen Song, Sandesh C.S. Nagamani, Dianne Nguyen, Ingo Grafe, Vernon Reid Sutton, Francis H. Gannon, Elda Munivez, Ming-Ming Jiang, Alyssa Tran, Maegen Wallace, Paul Esposito, Salma Musaad, Elizabeth Strudthoff, Sharon McGuire, Michele Thornton, Vinitha Shenava, Scott Rosenfeld, Shixia Huang, Roman Shypailo, Eric Orwoll, Brendan Lee

**Affiliations:** 1 Department of Molecular and Human Genetics, Baylor College of Medicine, Houston, Texas, USA.; 2 Texas Children’s Hospital, Houston, Texas, USA.; 3 Division of Endocrinology, Diabetes, and Bone Diseases, Department of Medicine and Center for Healthy Aging, University Hospital Carl Gustav Carus Dresden, Dresden, Germany, and Center for Regenerative Therapies Dresden, Technische Universität Dresden, Dresden, Germany.; 4 Pathology and Immunology and Orthopedic Surgery, Baylor College of Medicine, Houston, Texas, USA.; 5 Orthopaedic Surgery, University of Nebraska Medical Center, Children’s Hospital and Medical Center, Omaha, Nebraska, USA.; 6 Department of Pediatrics-Nutrition, Baylor College of Medicine, Houston, Texas, USA.; 7 US Department of Agriculture/Agricultural Research Service (USDA/ARS) Children’s Nutrition Research Center, Houston, Texas, USA.; 8 Department of Orthopedic Surgery,; 9 Departments of Molecular and Cellular Biology, Education, Innovation, and Technology, Dan L. Duncan Comprehensive Cancer Center, and Advanced Technology Cores/Office of Research, and; 10 USDA/ARS Children’s Nutrition Research Center, Department of Pediatrics, Baylor College of Medicine, Houston, Texas, USA.; 11 Department of Medicine, Bone and Mineral Unit, Oregon Health & Science University, Portland, Oregon, USA.

**Keywords:** Clinical Trials, Therapeutics, Bone disease, Drug therapy

## Abstract

**BACKGROUND:**

Currently, there is no disease-specific therapy for osteogenesis imperfecta (OI). Preclinical studies demonstrate that excessive TGF-β signaling is a pathogenic mechanism in OI. Here, we evaluated TGF-β signaling in children with OI and conducted a phase I clinical trial of TGF-β inhibition in adults with OI.

**METHODS:**

Histology and RNA-Seq were performed on bones obtained from children. Gene Ontology (GO) enrichment assay, gene set enrichment analysis (GSEA), and Ingenuity Pathway Analysis (IPA) were used to identify dysregulated pathways. Reverse-phase protein array, Western blot, and IHC were performed to evaluate protein expression. A phase I study of fresolimumab, a TGF-β neutralizing antibody, was conducted in 8 adults with OI. Safety and effects on bone remodeling markers and lumbar spine areal bone mineral density (LS aBMD) were assessed.

**RESULTS:**

OI bone demonstrated woven structure, increased osteocytes, high turnover, and reduced maturation. SMAD phosphorylation was the most significantly upregulated GO molecular event. GSEA identified the TGF-β pathway as the top activated signaling pathway, and IPA showed that TGF-β1 was the most significant activated upstream regulator mediating the global changes identified in OI bone. Treatment with fresolimumab was well-tolerated and associated with increases in LS aBMD in participants with OI type IV, whereas participants with OI type III and VIII had unchanged or decreased LS aBMD.

**CONCLUSION:**

Increased TGF-β signaling is a driver pathogenic mechanism in OI. Anti–TGF-β therapy could be a potential disease-specific therapy, with dose-dependent effects on bone mass and turnover.

**TRIAL REGISTRATION:**

ClinicalTrials.gov NCT03064074.

**FUNDING:**

Brittle Bone Disorders Consortium (U54AR068069), Clinical Translational Core of Baylor College of Medicine Intellectual and Developmental Disabilities Research Center (P50HD103555) from National Institute of Child Health and Human Development, USDA/ARS (cooperative agreement 58-6250-6-001), and Sanofi Genzyme.

## Introduction

Osteogenesis imperfecta (OI) is a genetically and phenotypically heterogeneous Mendelian disorder of connective tissue that has an estimated prevalence of 1 in 10,000 to 20,000 births. Skeletal manifestations of OI include low bone mass, bone fragility, recurrent fractures, scoliosis, and bone deformities. Extraskeletal manifestations include decreased muscle mass, muscle weakness, articular hyperlaxity, dentinogenesis imperfecta, hearing loss, and pulmonary disease (1–5). The management of OI typically involves a multidisciplinary approach that includes pharmacologic therapy to increase bone density, physical medicine and rehabilitation services to optimize function and mobility, audiology evaluations and hearing aids, dental and oral surgery for treatment of dentinogenesis imperfecta and malocclusion, screening for cardiovascular and pulmonary complications, and surgery for treatment of fractures, scoliosis, and bone deformities. Medical therapy for treatment of bone fragility is limited to repurposing of medications that are used to treat osteoporosis (6–17). Bisphosphonates (BPNs), a class of antiresorptive medications that decrease bone remodeling, have become the standard of care, especially in children with OI. In children, BPNs have been shown to have beneficial effects on areal and volumetric bone mineral density (aBMD and vBMD, respectively), quality of life, and, in some studies, fracture incidence and progression of scoliosis (8, 15, 18–20); however, given the heterogeneity of OI and the variability in the clinical study designs, the effects of BPN on these outcomes have been inconsistent (21, 22). In adults, the benefits and the consequences of long-term treatment with BPNs are less certain (6, 9, 23). Additionally, in a randomized trial, treatment with an anabolic agent, teriparatide, led to increase in aBMD and vBMD in adults with the mild form (OI type I) but not the moderate-to-severe forms of the disorder (OI types III and IV; ref. 16). Furthermore, none of these repurposed therapies address specific pathogenetic mechanism(s) in OI, and, hence, they have no effect on extraskeletal manifestations. Currently, there are no FDA-approved therapies for OI. Therefore, development of therapies targeting OI-specific mechanisms is of significant importance.

While pathogenic variants in multiple genes can cause various subtypes of OI, more than 90% of OI is caused by pathogenic variants in *COL1A1* or *COL2A1* and in genes encoding proteins that posttranslationally modify type I collagen (*CRTAP*, *PPIB*, and *LEPRE1*; refs. 3, 24). Using murine models of collagen-related forms of OI, *Col1a2^tm1.1Mcbr^* and *Crtap^–/–^* mice, which recapitulate the phenotypes in humans with OI, we have previously shown that excessive TGF-β signaling is a key driver of pathogenesis (25). The enhanced TGF-β–mediated signaling in mouse models of OI is, at least in part, due to the reduced binding affinity of TGF-β regulator decorin to the abnormal type I collagen, which thus affects TGF-β sequestration in the extracellular matrix (25). Importantly, we also showed that neutralizing TGF-β improves bone mass, bone biomechanical properties, as well as pulmonary abnormalities in these preclinical models (25). However, it is not known whether this mechanism is relevant in humans.

Here, we assessed the dysregulated pathways in bones from children with OI to understand whether increased TGF-β signaling is a potential target for therapy in humans. From a translational perspective, we also conducted a first-in-human phase I study in adults with OI to assess the safety of therapy with fresolimumab, a TGF-β neutralizing antibody, and explored its effects on bone remodeling and bone density.

## Results

### Disorganized woven bone and increased osteocyte density in bones from children with OI type III.

Bone fragments from tibias or femurs were collected from 10 children with OI type III (9 with glycine substitution mutations in *COL1A1* or *COL1A2* and 1 with valine deletion in *COL1A2*) and 4 children who were not affected by OI (Supplemental Table 1; supplemental material available online with this article; https://doi.org/10.1172/JCI152571DS1). Histologically, while the non-OI specimens contained mostly cortical bone, OI specimens contained both cortical and trabecular bone (Supplemental Figure 1). Morphological examination revealed that OI bones demonstrated disorganized Haversian system, with predominantly woven bone, compared with non-OI bones (Supplemental Figure 1). Consistent with earlier reports (26), osteocyte density was over 3-fold higher (686.44/mm^2^ vs. 221.94/mm^2^, *P =* 0.0056) in OI bone, with the lacunae appearing more spherical as compared with non-OI bone (Supplemental Figures 1 and 2).

### Global transcriptomic analysis demonstrates increased TGF-β signaling as a key dysregulated pathway in OI type III bone.

**Figure 1:**
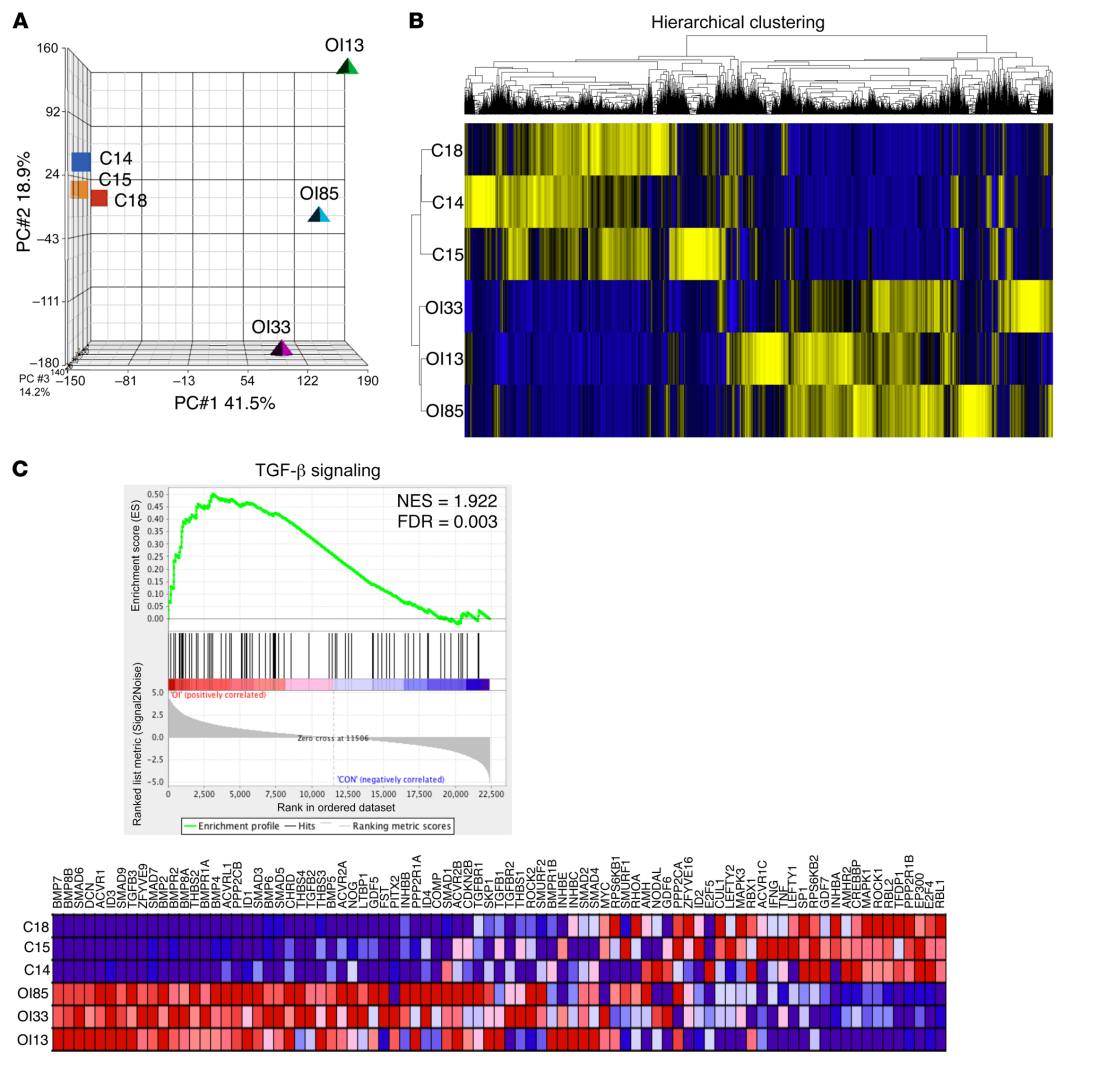
Transcriptomic and bioinformatics analyses demonstrate activation of TGF-β signaling in OI type III bone. (**A**) Principal component analysis (PCA) plot of transcriptomic data from non-OI and OI type III bones in 3 principal component dimensions. (**B**) Hierarchical clustering based on Euclidian distance using RPKM of all non-OI and OI type III bone data. Blue, downregulated; yellow, upregulated. (**C**) Gene set enrichment plot demonstrated activation of TGF-β signaling. C18, C14, and C15 represent 3 biologically distinct non-OI bone samples. OI85, OI33, and OI31 represent 3 biologically distinct OI type III bone samples. The expression pattern of genes involved in the TGF-β gene set in the analysis database is shown. NES, normalized enrichment score; FDR, false discovery rate. Blue, downregulated; red, upregulated.

To identify the key dysregulated pathways in human OI bones in an unbiased fashion, we performed RNA-Seq on OI type III and non-OI bones. To correlate RNA-Seq results with an orthogonal method, we validated a subset of differentially expressed genes from RNA-Seq using Nanostring on 1 OI sample (OI85) and 1 non-OI sample (C18). This analysis validated 155 genes by NanoString and demonstrated a consistency of 92% for expression fold change (Supplemental Table 2). Principal component analysis (PCA) of transcriptomic data revealed a clear separation between non-OI and OI bones (Figure 1A). The gene expression profile was more homogeneous in the non-OI bones compared with the OI bones. Hierarchical clustering of whole transcriptome reads per kilobase of transcript per million mapped reads (RPKM) showed distinct clusters between non-OI and OI bones, which indicated a change in overall molecular signature in OI (Figure 1B). Following differential gene expression analysis, GO enrichment analysis was performed. In GO biology processes, the significantly enriched skeletal-related functions captured the molecular signatures of OI, including increased bone formation, resorption, and remodeling, along with decreased bone trabecula and bone maturation. Consistently, in GO cellular processes, increased osteoblast and osteoclast differentiation was identified in OI. In GO signaling pathways, major bone remodeling pathways, including BMP/TGF-β, the parathyroid hormone pathway, WNT, and Notch signaling, were upregulated. Most interestingly, SMAD protein phosphorylation regulation was the most significantly upregulated pathway among them all (*P =* 1.78 × 10^–15^; Supplemental Table 3). These enrichment results are not only consistent with the known molecular pathological characteristics of OI bone, including molecular signatures of increased bone turnover and reduced bone maturation, but also reveal that a TGF-β downstream event, SMAD phosphorylation, was the most affected target in OI type III bone.

To independently explore signaling changes in OI bone, we next performed gene set enrichment analysis (GSEA) (27) using RPKM from non-OI and OI bones. GSEA identified TGF-β signaling as a top significantly activated pathway in OI (Figure 1C and Supplemental Table 4). Together, these results suggest that TGF-β signaling is a major and most consistently activated pathway in OI type III bone.

### TGF-β is an activated upstream regulator in OI type III bones.

To investigate whether the activation of the TGF-β pathway identified from transcriptomic analysis was due to regulation from TGF-β ligand(s), we utilized Ingenuity Pathway Analysis (IPA) for upstream regulator prediction based on 3722 genes that showed a significant change in expression (Supplemental Table 5). Among all potential upstream regulators identified, TGFB1 was the most activated (*Z* score = 4.28, *P =* 1.32 × 10^–14^; Table 1 and Supplemental Table 6). To ensure these transcriptomic findings induced changes at the protein level, we performed targeted proteomic reverse-phase protein array (RPPA). Among the 215 proteins examined (Supplemental Table 7), 29 proteins showed nominal statistically significant intensity change between OI and non-OI bones (Supplemental Table 8). Using these targeted-proteomic results, TGFB1 was identified as the most significantly activated upstream regulator by IPA (*Z* score = 2.17, *P =* 6.37 × 10^–12^; Table 1 and Supplemental Table 6).

### Increased SMAD2 phosphorylation in OI type III bone.

**Figure 2:**
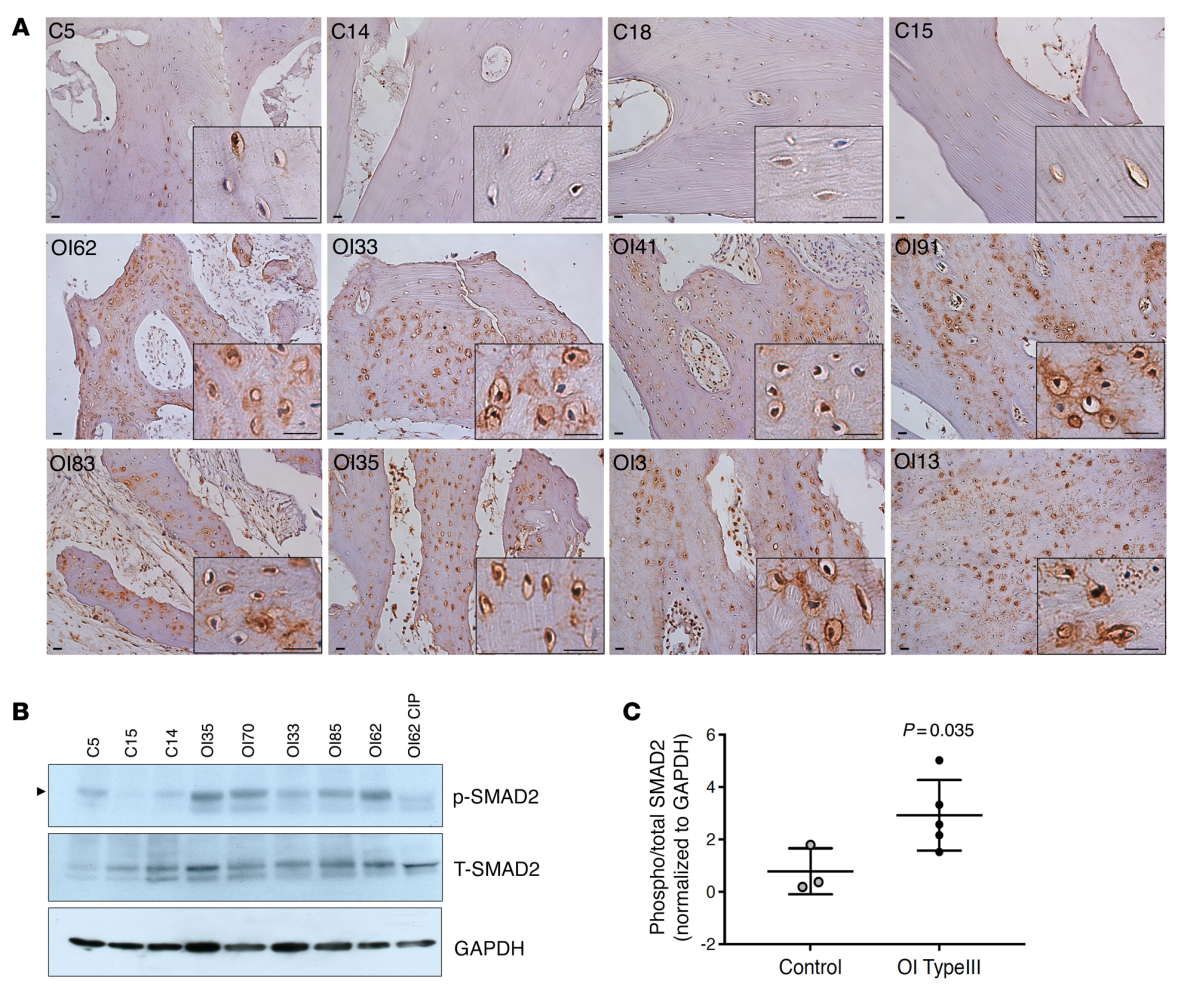
Increased phosphorylated SMAD2 in OI type III bone. (**A**) IHC staining of phosphorylated SMAD2 (pSMAD2) in non-OI and OI type III bone sections. Higher-magnification images are shown in black boxes on the bottom right. Increase in pSMAD2 signal was detected in all OI samples, especially in the osteocytes. Scale bar: 20 μm. (**B**) Western blot of p-SMAD2 and total SMAD2 (T-SMAD2) in protein extracted from non-OI and OI type III bone. A total of 50 μg protein was loaded. One OI bone sample (OI62) was treated with calf-intestinal alkaline phosphatase (CIP) to remove phosphorylation signal to serve as a negative control for accurate pSMAD2 signal (indicated by arrowhead). See complete unedited blots in the supplemental material. (**C**) Quantification of Western blot in **B**, showing the ratio of phosphorylated (phospho) versus total SMAD2. Data are shown as the mean ± SD. GAPDH was used as loading control. C, non-OI (*n =* 3); OI, OI type III (*n =* 5).

To conclusively show the increased TGF-β signaling in situ in OI type III bone, we performed IHC using antibody against phosphorylated SMAD2 (pSMAD2, TGF-β downstream target). Compared with non-OI bone, we found consistently increased pSMAD2 staining in OI type III bone (Figure 2A). Western blot (WB) using protein lysate demonstrated an increase in pSMAD2 in all OI bone samples examined (Figure 2B) and quantification demonstrated a significant increase in the pSMAD2/total SMAD2 ratio in OI bone (Figure 2C). All together, these results suggest that TGF-β activation is a major driving pathogenic mechanism in humans with OI.

### Fresolimumab as a therapeutic intervention in OI.

To translate these mechanistic findings, we conducted a phase I trial in adults with OI to evaluate the safety of fresolimumab, a human IgG4 κ monoclonal antibody that neutralizes all 3 TGF-β homodimers. To be consistent with the preclinical (25) and human data we generated, only adults (*n =* 8) with clinically moderate-to-severe OI caused by glycine substitution mutations in *COL1A1* or *COL1A2* or biallelic pathogenic variants in *CRTAP*, *PPIB*, or *LEPRE1*, i.e., structural mutations affecting type I collagen or mutations affecting the posttranslational modification machinery of type I collagen, were enrolled (Table 2). Four received a single administration of fresolimumab at a dose of 1 mg/kg body weight and 4 received a single dose of 4 mg/kg body weight. Treatment with fresolimumab was well-tolerated. There were no medication-related serious adverse events (AEs) in both cohorts, and no clinically significant laboratory changes were observed (Supplemental Figure 3). In the 1 mg/kg cohort, only 2 AEs were graded as being possibly/probably related to the study medication: nausea following administration of the drug and epistaxis. In the 4 mg/kg cohort, 7 AEs were graded as being possibly/probably related to the medication: malaise, headache, epistaxis, occult blood in urine, bleeding from skin scab, and corrected QT interval of 457 ms in 1 participant. A list of all AEs and their relationships to the study medication is provided in Supplemental Table 9.

**Figure 3:**
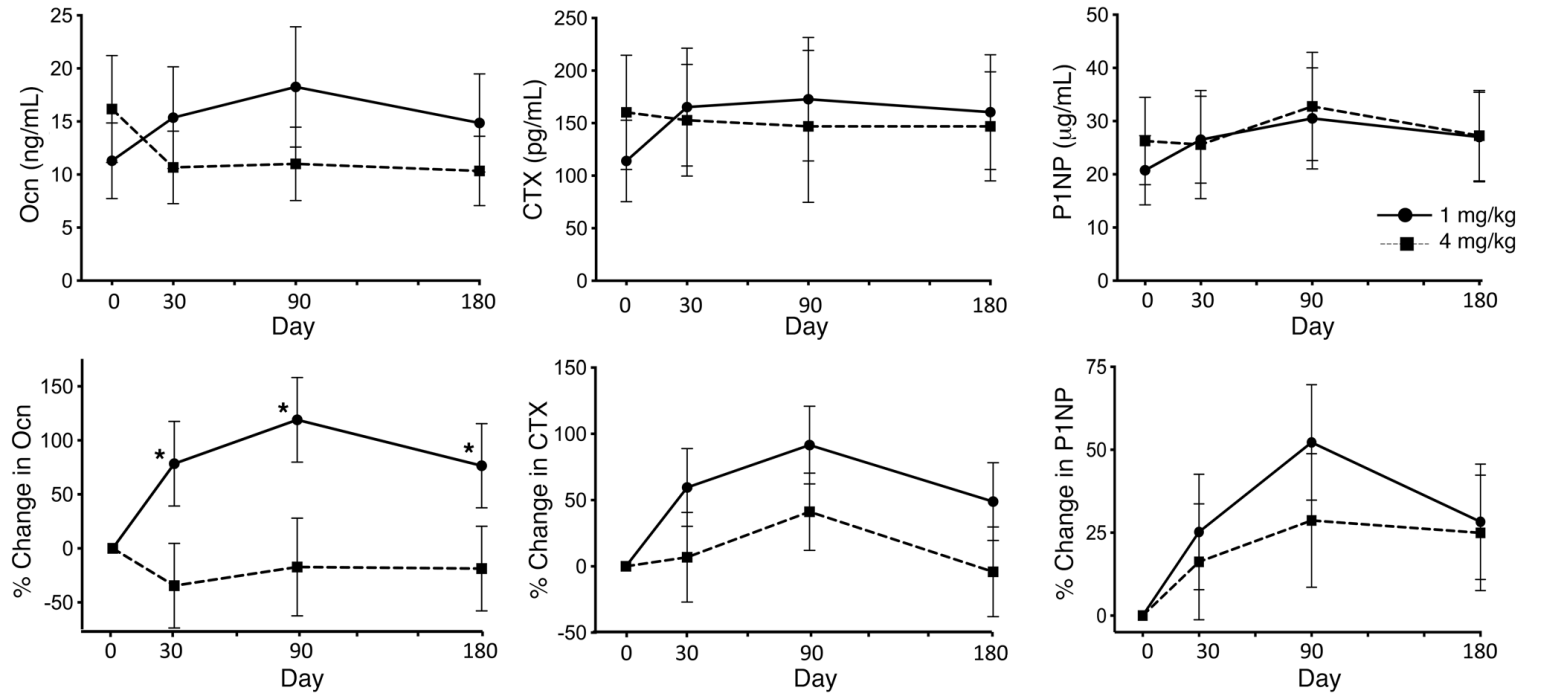
Effect of fresolimumab on bone turnover markers and bone density. The top row shows of serum levels of osteocalcin (Ocn), C-terminal telopeptide (CTX), and N-terminal propeptide of type 1 procollagen (P1NP) at each time point. The bottom row shows percentage changes in these markers of bone turnover as compared with baseline values. The solid lines with circles represent results for the 1 mg/kg dose cohort (*n =* 4), and the dotted lines with squares represent results for the 4 mg/kg dose cohort (*n =* 4). Data are shown as the mean ± SEM. *P < 0.05, GLM association.

**Figure 4:**
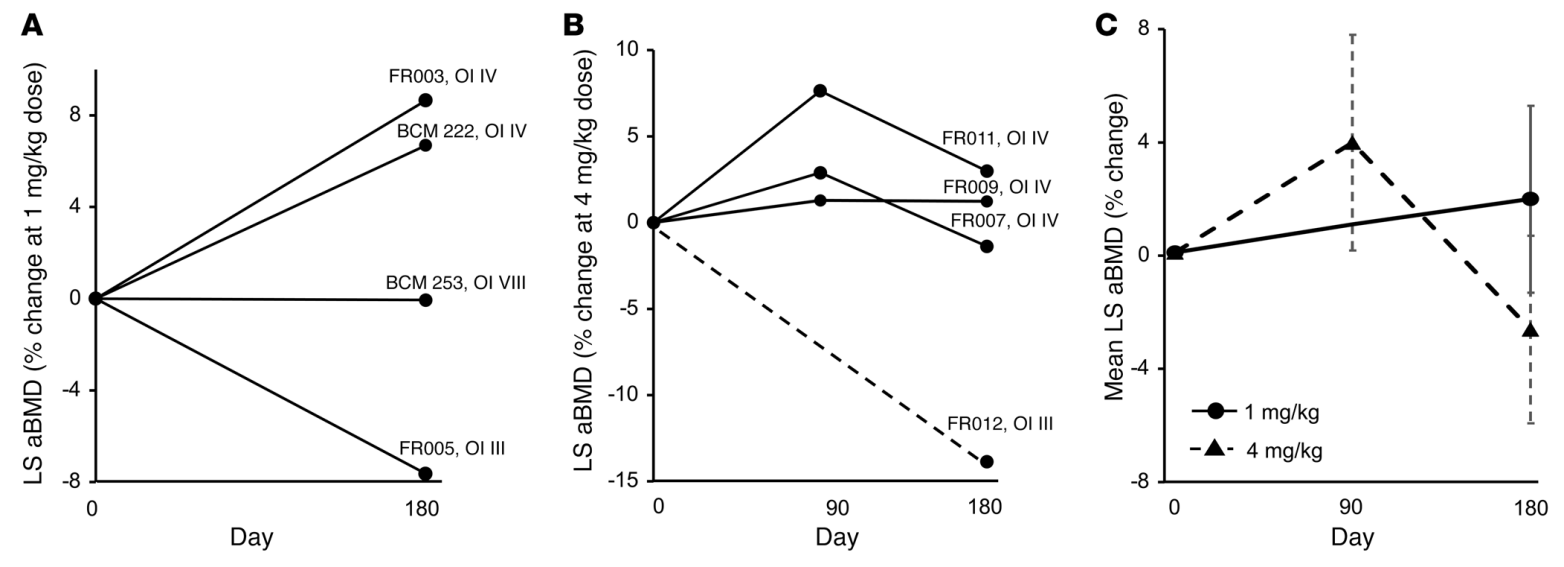
Effect of fresolimumab on LS aBMD. The percentage change in LS aBMD in (**A**) the 1 mg/kg dose cohort (*n =* 4) and (**B**) the 4 mg/kg dose cohort (*n =* 4). (**C**) Average aBMD changes at each time point based on dose. In **B**, aBMD could not be assessed in FR012 at the 90-day time point; therefore, the result is shown as a dotted line. In **C**, the solid lines represent results for the 1 mg/kg dose cohort, and the dotted lines represent results for the 4 mg/kg dose cohort. Data are shown as the mean ± SEM.

Even with the limited sample size, treatment with the 4 mg/kg dose was associated with a decrease in mean serum osteocalcin (Ocn) as compared with the 1 mg/kg dose. The percentage change in Ocn was significantly different between the 2 doses (*P =* 0.0045). Whereas there were no significant changes in C-terminal telopeptide (CTX) and N-terminal propeptide of type 1 procollagen (P1NP) between the 2 dose groups, similar to the results with Ocn, the percentage change of CTX from baseline was lower in the 4 mg/kg dose cohort (Figure 3). Lumbar spine aBMD (LS aBMD) results were analyzed by 2 central readers. There was a high degree of correlation (*r* = 0.995) as well as agreement between the 2 reads (Supplemental Figure 4). With the 1 mg/kg dose, 2 participants with OI type IV showed robust increases in LS aBMD at 6 months after treatment, while the individual with OI VIII did not demonstrate any change (Figure 4). The participant with OI type III who showed a drop in aBMD had severe scoliosis that posed technical difficulties in accurate assessment of aBMD. In the 4 mg/kg cohort, LS aBMD was assessed at days 90 and 180. Two participants with OI type IV had robust increases in aBMD by day 90 (Figure 4). The individual with OI type III who showed a sharp aBMD drop sustained a femur fracture, resulting in prolonged immobility; the aBMD at day 180 was measured at a remote facility making comparisons less than ideal.

## Discussion

Study of rare bone disorders is not only pivotal for developing effective therapies for these disorders, but also for identifying therapeutic targets for common bone diseases such as osteoporosis and osteosclerosis. For example, the study of genetic disorders with high and low bone mass has been key for developing therapies such as denosumab and romosozumab for osteoporosis (28, 29). In this study, we examined the global signaling abnormalities in OI, a Mendelian form of brittle bone disease. The findings and multiomic data from our study can be relevant to OI and other disorders of low bone mass.

Previous work in preclinical models has shown that increased TGF-β signaling is a pathogenetic mechanism in OI caused due to alterations in type I collagen and that inhibition of TGF-β could be of therapeutic benefit (25, 30–32). In order to facilitate clinical translation, we first profiled the transcriptome of human OI bone. GO pathway enrichment and GSEA independently identified activation of the TGF-β/SMAD signaling pathway as a top dysregulated event in bones from children with OI, and IPA analyses demonstrated that TGF-β1 was the most significantly activated upstream regulator responsible for the transcriptome changes. These transcriptomic changes were further corroborated at the protein level by RPPA, IHC, and WB analyses. The multiomic analysis of signaling changes as well as bioinformatic analyses demonstrated that excessive TGF-β activation is a key dysregulated pathway in human OI. We then assessed whether the increased TGF-β signaling can be targeted for therapy.

While repressing a pivotal pathway such as TGF-β in most tissues requires sustained pharmacological inhibition, the duration of human bone remodeling, which is approximately 3 months (33), allows for pharmacological inhibition at a single time point that may have effects beyond the terminal half-life of the therapeutic agent. This unique feature of bone offers potential safety advantages by decreasing cumulative dosage and administration frequency. Fresolimumab has been evaluated as a therapeutic option in myelofibrosis, advanced renal cell carcinoma, melanoma, metastatic breast cancer, idiopathic pulmonary fibrosis, systemic sclerosis, and focal segmental glomerulosclerosis (34–37). Whereas it is difficult to extrapolate the safety data from advanced cancer studies, one of the important safety signals from these trials was the development of reversible cutaneous keratoacanthomas, squamous cell carcinomas, and hyperkeratosis. Thus, in our clinical trial, we excluded individuals with preexisting history of basal cell carcinoma, squamous cell carcinoma, keratoacanthomas, actinic keratosis, and atypical moles. In clinical trials involving steroid-resistant primary focal segmental glomerulosclerosis and systemic sclerosis, increased risk for mucocutaneous bleeding has been observed (35, 36). Furthermore, epistaxis has also been reported in trials exploring inhibition of the TGF-β pathway in cancers, including STM 434, a soluble receptor ligand trap targeting activin A and bintrafusp α, a monoclonal antibody against programmed cell death ligand 1 (PD-L1) fused to an extracellular domain of TGF-βRII (38, 39). Thus, we excluded individuals with hemoglobin of less than 10 g/dL, platelet counts of less than 75,000/mm3, and prothrombin time and international normalized ratios more than 1.5 times the upper limit of normal. We also specifically assessed bleeding as a safety outcome. In this study, 2 individuals had mild epistaxis; and the platelet counts, prothrombin time, and international normalized ratios were normal in these individuals.

TGF-β is important for coupling of bone resorption and bone formation. At the 1 mg/kg dose, fresolimumab was associated with an increase in bone remodeling. However, treatment with the 4 mg/kg dose resulted in sustained suppression of bone turnover, as shown by plasma Ocn levels. The latter effect is consistent with murine studies that show neutralizing TGF-β results in a decrease in bone turnover and an increase in bone mass (25, 31). The effects on LS aBMD were more variable. Two participants with OI type IV had increases of 6.8% and 8.6% in LS aBMD with the 1 mg/kg dose. In the 4 mg/kg cohort, 1 participant had an increase of 7.6%, while 2 had increases of 2.9% and 1.3%, 3 months after infusion. These increases are higher than increases in LS aBMD seen after treatment with the anabolic agent teriparatide, which demonstrated a 2% increase at 6 months in individuals with mild but not severe OI (16). The increases are comparable to those seen after treatment with monthly high-dose setrusumab, which was associated with a 5.4% in LS aBMD increase over 6-month trial period in OI types III and type IV (40, 41). Two participants with OI type III (FR005 and FR012) had decreases in aBMD. FR005 had severe scoliosis that affected measurement accuracy. FR012 had prolonged immobility due to a fracture. Furthermore, due to the travel restrictions owing to the ongoing COVID-19 pandemic, dual-energy X-ray absorptiometry (DXA) assessment was performed at a remote facility. Though a comparable densitometer was used, such comparisons are less than ideal.

Genotype and clinical severity are likely to have an influence on the response to therapy. Recent preclinical studies have shown that higher and more frequent dosing of anti–TGF-β therapy has a more significant effect on “normalizing” the coupling of bone resorption and bone formation, resulting in decreased bone remodeling, increased bone mineral density, and improved biomechanical properties (31). Furthermore, whereas mild and moderately severe murine models of collagen-related OI (G610C *Col1a2^tm1.1Mcbr^* and Crtap^–/–^) have shown a response to anti–TGF-β treatment, such response has not been observed in a more severe model (*Col1a1^Jrt/+^*) that has features of Ehlers-Danlos syndrome and OI (30). These preclinical observations, together with the human data here, suggest that there may be a differential dose requirement for different levels of OI severity, perhaps, in part, related to the magnitude of TGF-β dysregulation in these states. Additionally, the differential response(s) to treatment may also be influenced by the crosstalk and ligand competition between TGF-β and other related signaling pathways, such as BMP signaling. It has been shown that TGF-β/SMAD2/3-activating ligands could antagonize SMAD1/5/8-activating ligand (42). Thus, increased TGF-β signaling may also modulate signaling by this closely related pathway.

The results of our study have to be taken in the context of their strengths and limitations. The strengths of the study include the following. (a) This is the first study to our knowledge to comprehensively characterize the signaling changes in bones from humans with OI. (b) The multiomic approach allowed for unbiased evaluation of signaling changes. (c) The clinical study leveraged the infrastructure of the NIH Rare Diseases Clinical Research Network and, thus, the safety and efficacy data points were robustly collected. The limitations of the study include the following. (a) We used bones obtained from children with and without OI. Children without OI had other medical diagnosis for which surgery was performed, i.e., acetabular dysplasia, bilateral subluxation of hip, and cerebral palsy. The average age of children without OI was higher than that of children with OI. All children with OI had received BPNs. It is possible that age, diet, BPN use, medical diagnosis, and use of other medications could have affected the transcriptomic signature in bone. Given the exceedingly rare situations in which healthy children get skeletal surgeries, it was not possible to obtain true age-matched healthy control bone. However, the fact that the transcriptomes of 3 non-OI samples clustered together on PCA provides support to the notion that transcriptome profiles were similar in these non-OI specimens and that they were clearly distinct from OI specimens. (b) The sample size for the transcriptomic analysis was relatively small due to challenges associated with obtaining surgical samples and the size of biosamples. We could not procure high-quality RNA and protein from all samples that we received. It is possible that the analyses could have missed uncovering effects associated with hormones, age-related gene expression patterns, and other potential regulators due insufficient power. (c) Bone turnover markers in individuals with OI can be influenced by many factors, including intercurrent fractures and history of previous treatment with BPN. One participant was BPN-naive, 4 participants had not received any BPN 5 years prior to enrollment, and 3 participants had received intravenous BPN 14 months before enrollment. Whereas there was no obvious discernible relationship between the timing of BPN administration and the effect on bone turnover markers or aBMD in this study, given the small numbers of participants, we cannot make definitive conclusions regarding the effect of previous treatment on remodeling changes associated with fresolimumab. (d) The wide age range of participants introduced confounders, such as age and hormonal status, that can affect bone turnover and density. While we tried to account for the repeated measures in participants and have modeled the observed change using a generalized linear model, with the small sample size, we cannot dissect the contributions of these confounders to the overall results.

In conclusion, our study shows excessive TGF-β signaling in human OI bone and that inhibition of TGF-β could be a valuable molecular mechanism–specific therapeutic strategy. The dosing may need to be matched with magnitude of TGF-β dysregulation. Our findings could also further contribute to the understanding of low bone density in other Mendelian collagen diseases.

## Methods

### Human bone sample collection and processing.

Bone samples from children with OI and children unaffected by OI were obtained when these children were undergoing skeletal surgery for a medical indication. The fragments of bone removed during surgery, which otherwise would have been discarded, were collected and processed. Demographic information as well as analysis conducted on each sample are provided in Supplemental Table 1. Bone specimens were processed in a liquid nitrogen-based environment, as described in Supplemental Figure 5, using a previously reported protocol (43). In short, to extract RNA and protein, fragments of bone were pulverized in a liquid nitrogen-based environment using a power drill. A 2–3 mm^3^ fragment right next to the pulverized area was dissected from each specimen for histology.

### Histology and osteocyte density analysis.

The processed bone specimens were fixed in 4% paraformaldehyde (MilliporeSigma) for 48 hours and decalcified in10% EDTA (MilliporeSigma) for 14 days at 4°C. Paraffin sections were stained with hematoxylin and eosin for morphological and osteocyte number analysis. Osteocyte density was calculated using Bioquant osteo (BIOQUANT Image Analysis Corporation).

### RNA extraction, RNA-Seq, and data analysis.

Total RNA from pulverized bone was extracted using TRIzol (Thermo Fisher Scientific) and further purified by lithium chloride precipitation. RNA quality and quantity were measured by Bioanalyzer (Agilent Technologies). 250 ng total RNA was used for TureSeq Stranded mRNA library preparation (Illumina) with ERCC spike-in (Thermo Fisher Scientific) applied according to manufacturer’s instructions. 22 pM of equimolarly pooled library was loaded onto 1 lane of a high-output v4 flow cell for bridge amplification using the Illumina cBot machine. A paired-end 100 cycle run was used to sequence the flow cell on a HiSeq 2500 Sequencing System in High Output Mode with v4 chemistry (FC-401-4003, Illumina). PhiX Control v3 adapter-ligated library (Illumina) was spiked-in at 2% by weight to ensure balanced diversity and to monitor clustering and sequencing performance. An average of 42.5 million paired-end reads was generated for each sample. The alignment was performed using HISAT2 through Genialis (https://www.genialis.com) with hg19-ERCC as reference. Normalization, differential expression, hierarchical clustering, and GO analysis were then performed using RNA-Seq analysis pipeline in Partek Genomics Suite (Partek). Statistical significance was determined by 2-way ANOVA, which is built into the Partek Genomics Suite RNA-Seq analysis pipeline. The significantly differential expressed genes (fold change >2 and false discovery rate < 0.05) were then loaded into IPA (Qiagen) for upstream regulator prediction. The GSEA program (Broad Institute) for pathway enrichment analysis was used according to the developer’s instructions.

### RNA-Seq validation by NanoString.

NanoString human WNT nCounter panel was run using 1 OI type III and 1 non-OI sample with RNA-Seq data for the validation of gene expression fold change detected. The analysis was performed using nSolver under regular module. Genes with read count below 20 were considered as background and were removed from further analysis. Total 155 genes were validated. The consistency was determined by the percentage of genes that showed the same change in expression directions.

### Protein extraction, RPPA, and WB.

Protein was extracted from pulverized bone by using lysis buffer overnight in 4°C (250 mM EDTA, 6 M guanidine-HCl, 50 mM Tris-HCl, pH 7.4). The extract was concentrated by methanol-H_2_O-chloroform precipitation and dissolved in 4% SDS buffer (4% SDS, 50 mM Tris, 5 mM EDTA, pH 7.4) for WB or dissolved at 0.5 mg/ml in RPPA lysis buffer containing SDS sample buffer and 0.25% β-mercaptoethanol. The protein concentration was measured using a BCA kit (Thermo Fisher Scientific). The RPPA was conducted by a core facility at Baylor College of Medicine (https://www.bcm.edu/academic-centers/dan-l-duncan-comprehensive-cancer-center/research/cancer-shared-resources/reverse-phase-protein-array) using a standardized protocol (44). In total, 4 non-OI control and 8 OI type III bone samples were included in the experiment. Each sample was assayed in 3 technical replicates to account for technical variation. The protein expression intensity of each biological sample was first derived from the calculated the mean intensity of the technical triplicate of each biological sample. The average expression intensity of the non-OI control group and OI type III group were then calculated from the mean of each sample’s protein expression intensity in the group. The 2-tailed Student’s *t* test was used to determine the statistical significance between non-OI control and OI type III. A nominal *P* value of 0.05 was used to determine significance. For WB, 50 μg total protein was used for loading. After separation by SDS-PAGE gel and transfer to PVDF membrane (MilliporeSigma), 5% milk was used to block the membrane, followed by overnight incubation with primary antibody (phospho-SMAD2 (3108S, Cell Signaling), SMAD2 (5339S, Cell Signaling), and GAPDH (G9295, MilliporeSigma) at 4°C. Signals were captured by films.

### IHC.

After deparaffinization, sections were incubated at 37°C with 0.05% trypsin for antigen retrieval following 3% hydrogen peroxide treatment. After blocking with 5% normal goat serum, sections were incubated with primary antibodies of phospho-SMAD2 (44-244G, Thermo Fisher Scientific) overnight at 4°C as per manufacture’s instructions. Anti-rabbit secondary antibody (Vectastain ABC system, Vector Laboratories) was applied, and blots were developed using 0.1% 3, 39-diaminobenzidine. Image were taken by Axioplan 2 imaging (ZEISS).

### Phase I clinical study design.

**Figure 5:**
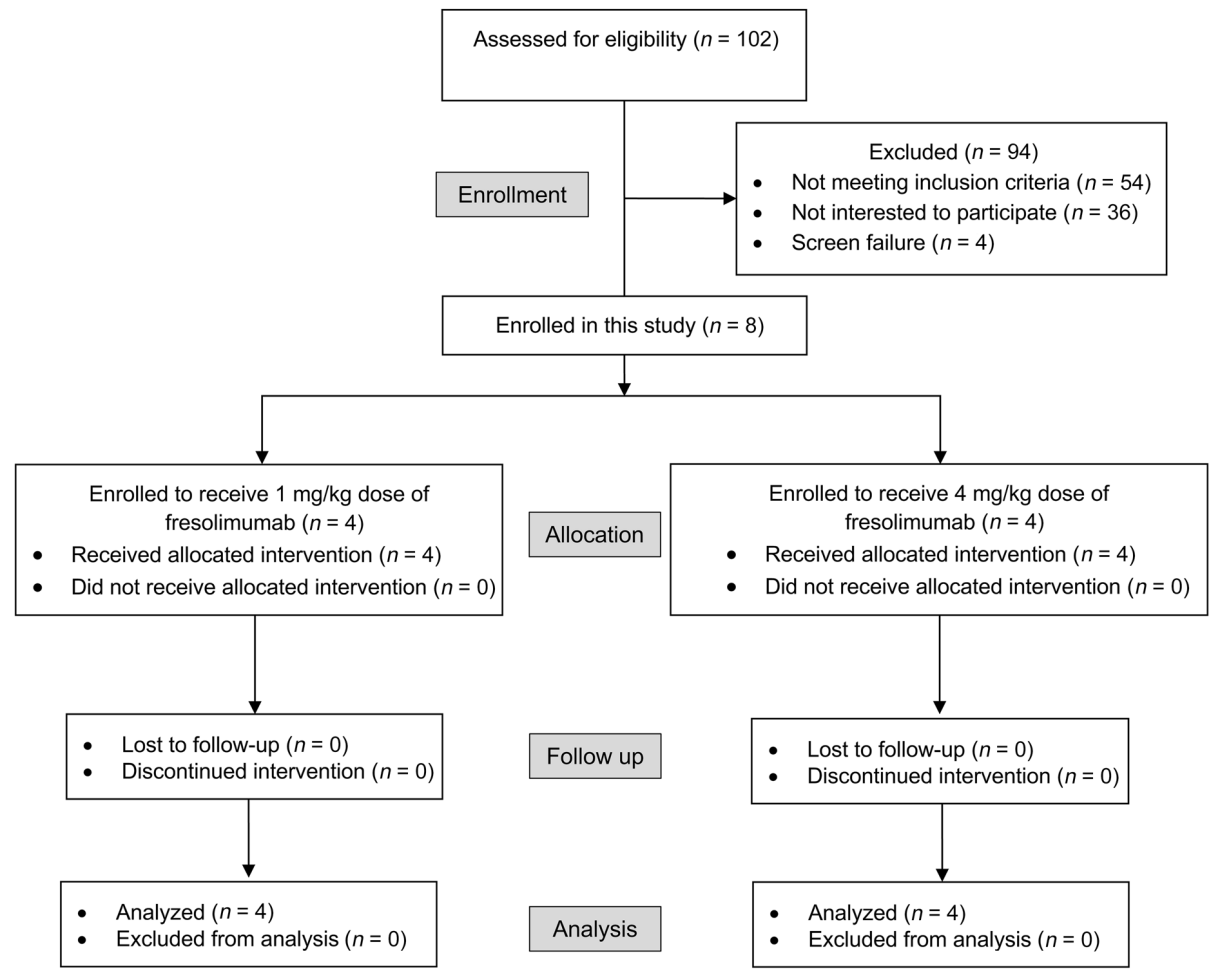
CONSORT flow diagram depicting screening, enrollment, and follow up of participants in the trial.

A phase I, dose-escalating clinical trial evaluating fresolimumab in adults with moderate-to-severe forms of OI was conducted as a part of the NIH Rare Disease Clinical Research Network’s Brittle Bone Disorders Consortium (BBDC) (ClinicalTrials.gov NCT03064074). While the study was also supported by a research agreement from Sanofi Genzyme, the design and conduct of the study were performed completely by the investigators. Stage 1 of the study involved a single infusion of fresolimumab (1 mg/kg body weight and 4 mg/kg body weight; *n =* 4 in each dose cohort) (Figure 5). The 2 doses were chosen based on available safety and tolerability data from previous phase I and phase II clinical studies of fresolimumab in humans (34–37). The study procedures outlined here were conducted between November 2017 and September 2020. First, 4 participants were enrolled in the 1 mg/kg dose cohort. After completion of this stage, the data safety monitoring board reviewed the safety data and approved enrollment of 4 individuals in the 4 mg/kg dose cohort. The total follow-up period in each dose cohort was 6 months. The primary outcome measure was safety of a single administration of fresolimumab. The secondary outcomes were the effects of fresolimumab on bone turnover markers Ocn, CTX, and P1NP in blood and on LS aBMD, as assessed by DXA. Individuals over 18 years of age with a diagnosis of moderate-to-severe OI based on having sustained 20 or more fractures during their lifetime and a glycine substitution mutation in *COL1A1* or *COL1A2* or biallelic pathogenic variants in *CRTAP*, *PPIB*, or *LEPRE1* were enrolled. Exclusion criteria were (a) instrumentation at the LS and both hips precluding assessment of aBMD, (b) long bone fractures 3 months prior to screening, (c) treatment with oral BPN within 6 months of screening or with intravenous BPN or teriparatide within 12 months of screening, (d) expected skeletal surgery, and (e) having characteristics that could affect safety, such as autoimmune disease, tuberculosis, history of cancer or precancerous lesions, cardiac valvular disease, and bleeding diathesis. Markers of bone turnover were measured by CLIA- and CAP-certified laboratories. DXA scans were performed using a Hologic Discovery densitometer at Texas Children’s Hospital. The scans were read by 2 central readers who were blinded to study procedures. All the data were entered onto an electronic data capture platform managed by the Data Management and Coordinating Center of the Rare Diseases Clinical Research Network.

### Bone turnover markers and LS aBMD analysis.

We tested the association between each bone turnover marker, i.e, Ocn, CTX, and P1NP, and time, dose, and the time-by-dose interaction using generalized linear mixed models. The log-normal values of bone turnover outcomes were used, and autoregressive covariance structure was specified to account for the repeated measures within an individual. To understand the overall effects of therapy on bone remodeling, the percentage change from baseline of each bone turnover marker at various time points was calculated. Percent changes were analyzed using a general linear model while adjusting for time and dose. Comparisons of mean percentage change were adjusted using the Tukey method.

The scans were read by 2 central readers who were blinded to study procedures. Correlation between the 2 reads was analyzed using standard correlation analysis. Agreement between the 2 reads was performed using Bland-Altman analysis. To understand the overall effects of therapy on LS aBMD, percentage change from baseline at various time points were calculated. Percentages of change were analyzed using a general linear model while adjusting for time and dose. The comparisons of mean percentage change were adjusted using the Tukey method.

### Statistics.

The statistical methods and the significance criteria for gene expression analysis, protein level analysis, and trial outcomes evaluations are listed in corresponding individual method sections above. They include FDR, 2-tailed Student’s *t* test, and 2-way ANOVA. A nominal *P* value of 0.05 was used to determine significance.

### Study approval.

For the gene and protein expression studies, bone samples from children with OI and children unaffected by OI were obtained under a protocol approved by the Baylor College of Medicine IRB. For the phase I clinical study, the study protocol was reviewed and approved by the NIH and the Baylor College of Medicine IRB. The study was monitored by a data safety monitoring board organized by the National Institute of Arthritis and Musculoskeletal and Skin Diseases. Informed consent was obtained from the parents or legal guardians prior to collection of all samples.

## Author contributions

Patient recruitment, clinical information collection, specimen collection, specimen shipment, and study coordination for the RNA and protein studies were done by IS, SCSN, IG, AT, MW, PE, ES, S McGuire, MT, VS, SR, and EO. Experimental design and interpretation were done by IS, SCSN, IG, and BL. Histology was performed by EM and MJ. The histological examination was performed by FHG. Bone specimen processing, osteocyte density analysis, RNA and protein extraction, transcriptome and RPPA bioinformatic analyses, WB, and IHC were done by IS. RPPA experiments were performed by SH. The phase I clinical trial was conducted by SCSN, DN, VRS, and BL. DXA analysis were done by RS and EO. Statistical analyses were performed by SCSN and S Musaad. The manuscript was written by IS, SCSN, and BL, with input from all authors. IS and SCSN contributed equally to the study; authorship order is based on the fact that IS led the basic science aspect of the project while SCSN led the clinical trial aspect of the project.

## Supplementary Material

Supplemental data

ICMJE disclosure forms

Supplemental tables 1-9

## Figures and Tables

**Table 2:**
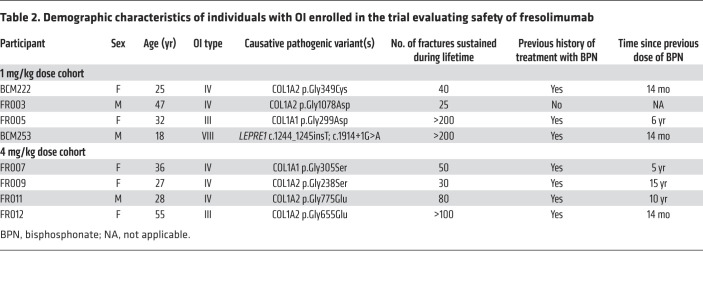
Demographic characteristics of individuals with OI enrolled in the trial evaluating safety of fresolimumab

**Table 1:**
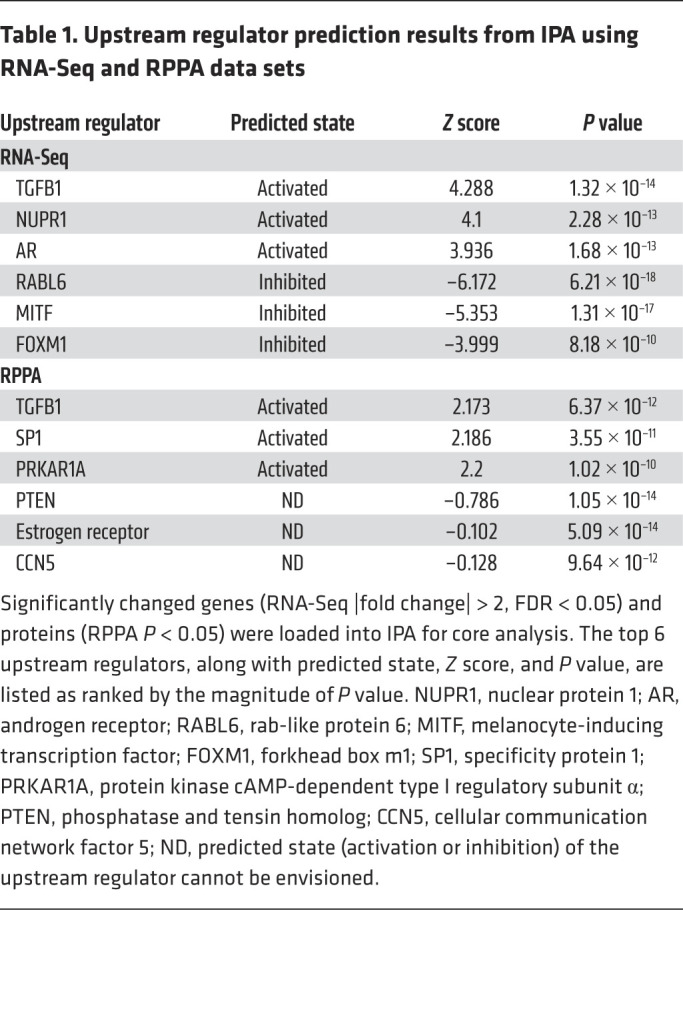
Upstream regulator prediction results from IPA using RNA-Seq and RPPA data sets

## Version 1 02/03/2022

In-Press Preview

## Version 2 04/01/2022

Electronic publication

## References

[1] Marini JC (2017). Osteogenesis imperfecta. Nat Rev Dis Primers.

[2] Marom R (2016). Pharmacological and biological therapeutic strategies for osteogenesis imperfecta. Am J Med Genet C Semin Med Genet.

[3] Patel RM (2015). A cross-sectional multicenter study of osteogenesis imperfecta in North America - results from the linked clinical research centers. Clin Genet.

[4] Rossi V (2019). Osteogenesis imperfecta: advancements in genetics and treatment. Curr Opin Pediatr.

[5] Tam A (2018). A multicenter study to evaluate pulmonary function in osteogenesis imperfecta. Clin Genet.

[6] Adami S (2003). Intravenous neridronate in adults with osteogenesis imperfecta. J Bone Miner Res.

[7] Bishop N (2010). Characterising and treating osteogenesis imperfecta. Early Hum Dev.

[8] Bishop N (2013). Risedronate in children with osteogenesis imperfecta: a randomised, double-blind, placebo-controlled trial. Lancet.

[9] Chevrel G (2006). Effects of oral alendronate on BMD in adult patients with osteogenesis imperfecta: a 3-year randomized placebo-controlled trial. J Bone Miner Res.

[10] DiMeglio LA, Peacock M (2006). Two-year clinical trial of oral alendronate versus intravenous pamidronate in children with osteogenesis imperfecta. J Bone Miner Res.

[11] Gatti D (2005). Intravenous neridronate in children with osteogenesis imperfecta: a randomized controlled study. J Bone Miner Res.

[12] Gatti D (2013). Teriparatide treatment in adult patients with osteogenesis imperfecta type I. Calcif Tissue Int.

[13] Glorieux FH (1998). Cyclic administration of pamidronate in children with severe osteogenesis imperfecta. N Engl J Med.

[14] Rauch F (2009). Risedronate in the treatment of mild pediatric osteogenesis imperfecta: a randomized placebo-controlled study. J Bone Miner Res.

[15] Rauch F (2003). Bone mass, size, and density in children and adolescents with osteogenesis imperfecta: effect of intravenous pamidronate therapy. J Bone Miner Res.

[16] Orwoll ES (2014). Evaluation of teriparatide treatment in adults with osteogenesis imperfecta. J Clin Invest.

[17] Hoyer-Kuhn H (2016). Safety and efficacy of denosumab in children with osteogenesis imperfecta — a first prospective trial. J Musculoskelet Neuronal Interact.

[18] Bains JS (2019). A multicenter observational cohort study to evaluate the effects of bisphosphonate exposure on bone mineral density and other health outcomes in osteogenesis imperfecta. JBMR Plus.

[19] Anissipour AK (2014). Behavior of scoliosis during growth in children with osteogenesis imperfecta. J Bone Joint Surg Am.

[20] Rauch F (2007). Intracortical remodeling during human bone development — a histomorphometric study. Bone.

[21] Dwan K (2014). Bisphosphonate therapy for osteogenesis imperfecta. Cochrane Database Syst Rev.

[22] Dwan K (2016). Bisphosphonate therapy for osteogenesis imperfecta. Cochrane Database Syst Rev.

[23] Shi CG (2016). Efficacy of bisphosphonates on bone mineral density and fracture rate in patients with osteogenesis imperfecta: a systematic review and meta-analysis. Am J Ther.

[24] Lim J (2017). Genetic causes and mechanisms of Osteogenesis Imperfecta. Bone.

[25] Grafe I (2014). Excessive transforming growth factor-β signaling is a common mechanism in osteogenesis imperfecta. Nat Med.

[26] Nijhuis WH (2019). Current concepts in osteogenesis imperfecta: bone structure, biomechanics and medical management. J Child Orthop.

[27] Subramanian A (2005). Gene set enrichment analysis: a knowledge-based approach for interpreting genome-wide expression profiles. Proc Natl Acad Sci U S A.

[28] Hannan FM (2019). Genetic approaches to metabolic bone diseases. Br J Clin Pharmacol.

[29] Rachner TD (2011). Osteoporosis: now and the future. Lancet.

[30] Tauer JT (2019). Effect of anti-TGF-β treatment in a mouse model of severe osteogenesis imperfecta. J Bone Miner Res.

[31] Greene B (2021). Inhibition of TGF-β increases bone volume and strengthin a mouse model of osteogenesis imperfecta. JBMR Plus.

[32] Kaupp S (2020). Combination therapy in the Col1a2(G610C) mouse model of Osteogenesis Imperfecta reveals an additive effect of enhancing LRP5 signaling and inhibiting TGFbeta signaling on trabecular bone but not on cortical bone. Bone.

[33] Langdahl B (2016). Bone modeling and remodeling: potential as therapeutic targets for the treatment of osteoporosis. Ther Adv Musculoskelet Dis.

[34] Morris JC (2014). Phase I study of GC1008 (fresolimumab): a human anti-transforming growth factor-beta (TGFbeta) monoclonal antibody in patients with advanced malignant melanoma or renal cell carcinoma. PLoS One.

[35] Vincenti F (2017). A phase 2, double-blind, placebo-controlled, randomized study of fresolimumab in patients with steroid-resistant primary focal segmental glomerulosclerosis. Kidney Int Rep.

[36] Rice LM (2015). Fresolimumab treatment decreases biomarkers and improves clinical symptoms in systemic sclerosis patients. J Clin Invest.

[37] Formenti SC (2018). Focal irradiation and systemic TGFβ blockade in metastatic breast cancer. Clin Cancer Res.

[38] Strauss J (2020). Bintrafusp alfa, a bifunctional fusion protein targeting TGF-β and PD-L1, in patients with human papillomavirus-associated malignancies. J Immunother Cancer.

[39] Tao JJ (2019). First-in-human phase I study of the activin A inhibitor, STM 434, in patients with granulosa cell ovarian cancer and other advanced solid tumors. Clin Cancer Res.

[40] Glorieux FH (2017). BPS804 anti-sclerostin antibody in adults with moderate osteogenesis imperfecta: results of a randomized phase 2a trial. J Bone Miner Res.

[41] Eric T (2021). Proceedings of the 2020 Rare Bone Disease Working Group. JBMR Plus.

[42] Martinez-Hackert E (2021). Receptor binding competition: A paradigm for regulating TGF-β family action. Cytokine Growth Factor Rev.

[43] Chou CH (2013). Direct assessment of articular cartilage and underlying subchondral bone reveals a progressive gene expression change in human osteoarthritic knees. Osteoarthritis Cartilage.

[44] Bu W (2019). Mammary precancerous stem and non-stem cells evolve into cancers of distinct subtypes. Cancer Res.

