# Supplementary material for: Targeting TGF-β for treatment of osteogenesis imperfecta
Source: J Clin Invest. 2022 Apr 1;132(7):e152571. doi: 10.1172/JCI152571 (PMC8970679; doi:10.1172/JCI152571)

Supplementary Figures and Tables

Figure S1

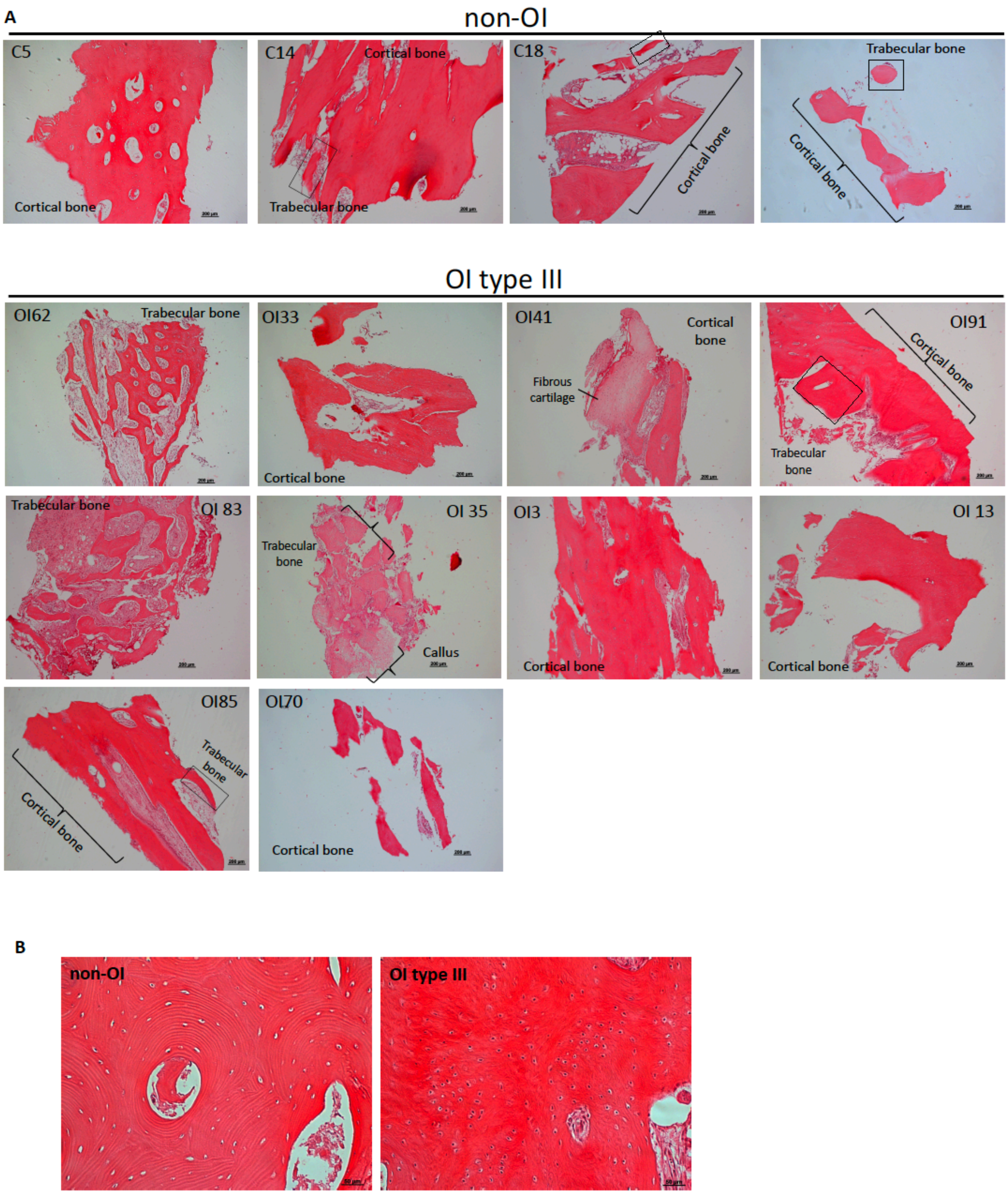

Supplementary Figure 1. Histology of bones from children with OI type III and children without OI. (A) Whole images of H&E stain sections. The majority of specimens from children without OI were cortical bone. The OI type III specimens were more heterogeneous. Whereas the majority were cortical bone, two specimens (OI62 and OI83) contained only trabecular bone. One specimen (OI41) had fibrous cartilage and one specimen (OI35) contained callus and trabecular bone. Scale bar: 200 μm. **(B)** Representative higher magnification (10X) of H&E stain images of non-OI and OI type III cortical bones. In the non-OI bone, well-organized Haversian canal system was observed while the OI type III bone had a more woven appearance with a less organized Haversian canal system. OI bone demonstrated more osteocytes with a spherical shape. Scale bar: 50 μm.

Figure S2

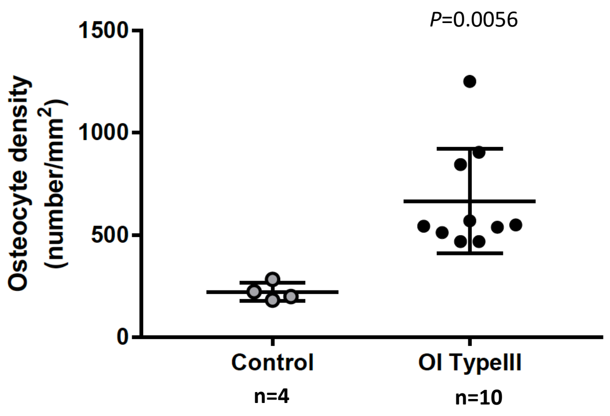

Supplementary Figure 2. Increased osteocyte density in OI type III bone. Quantification of osteocyte density in non-OI (n=4) and OI type III (n=10) bones. Each dot represents osteocyte density observed in one bone sample. Mean and standard deviation are depicted.

Figure S3

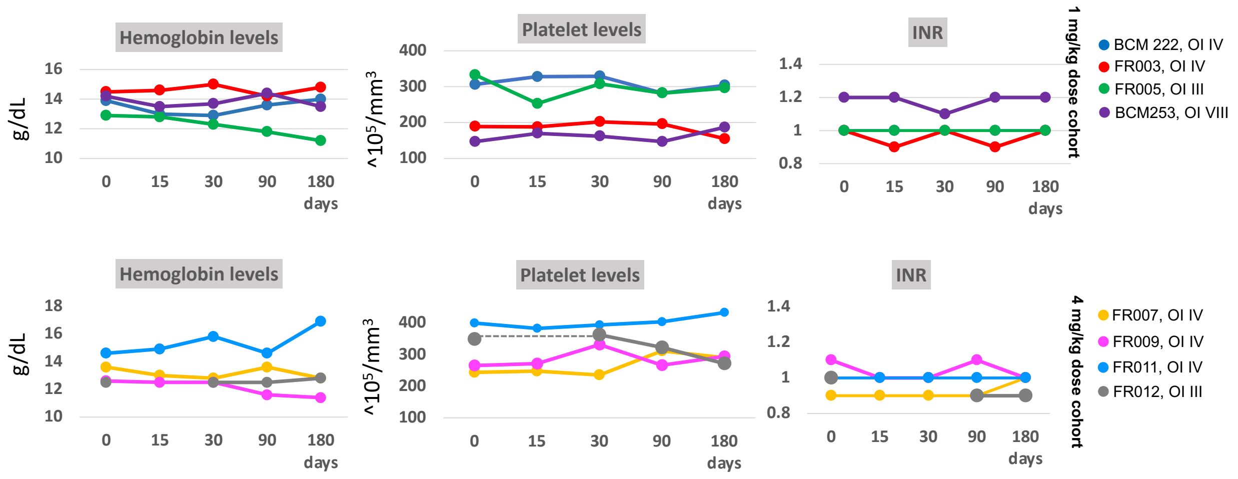

Supplementary Figure 3. Hematological safety data from the trial evaluating safety of fresolimumab in adults with OI. A drop in hemoglobin was noted in two participants (FR005 and FR009), both graded as mild. These two individuals had epistaxis that was graded as being related to the study medication and menstrual bleeding that was not graded as being unrelated to study medication. The platelet count and the INR were within normal limits.

Figure S4

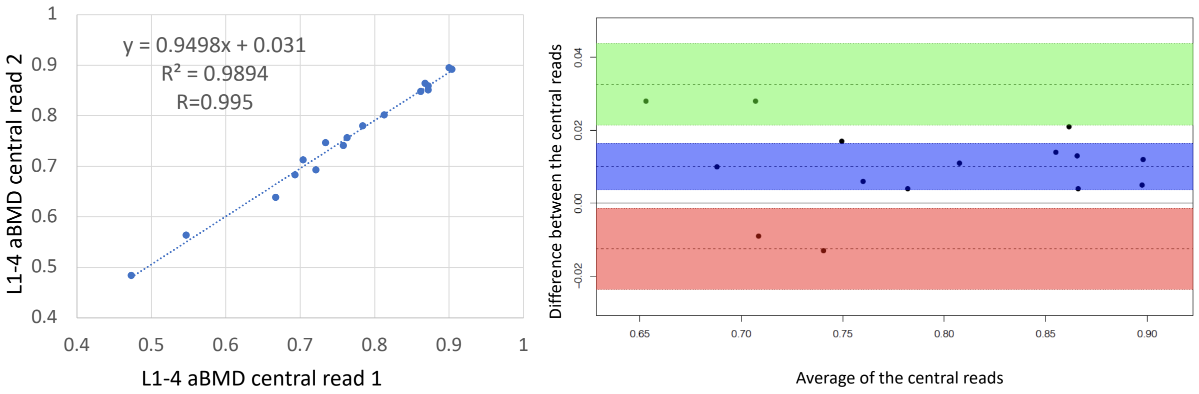

Supplementary Figure 4. Correlation and agreement between the two central reads for LS aBMD. The LS aBMD was read by two independent readers who were blinded to the trial design. There was a strong correlation between the two reads (R=0.995). A Bland-Altman plot showed a high degree of agreement between the two reads. The mean difference, upper limit of agreement, lower limit of agreement, and the confidence intervals are depicted in blue, green, and red, respectively.

Figure S5

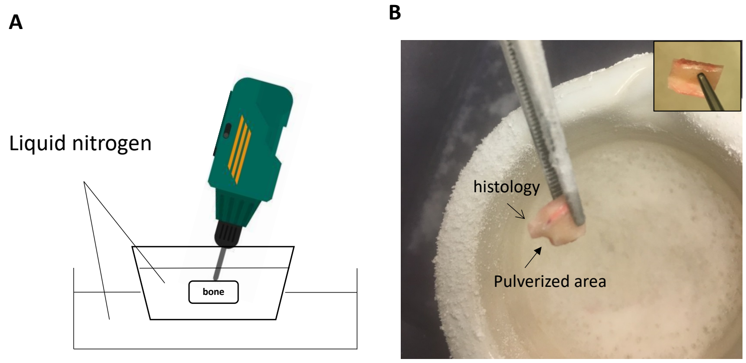

Supplementary Figure 5. Human bone specimen processing for histology, RNA, and protein studies. (A) An illustration of bone specimen pulverization environment showing that bone specimen was immersed in double-layered liquid nitrogen and pulverized by electric-powered drill. **(B)** A representation of a bone specimen prior to processing is shown at the upper right corner. After pulverization, bone powder at the bottom of a pestle was collected in liquid nitrogen. A 2-3 mm^3 area immediately next to the pulverized region was processed for histology and immunochemistry.

Supplementary Table Legends

Supplementary Table 1. Characteristics of children from whom bone was collected for analysis and analyses conducted on bone samples. Bone specimens were collected from femur or tibia. The experimental assays performed on each individual specimen are outlined. Given the limitations of the size of bone specimens received and the quality as well as quantity of RNA and protein extracted from these samples, every experimental analysis could not be conducted on all samples. HE: hematoxylin and eosin stain; IHC: immunohistochemistry; WB: Western blot; RNASeq: transcriptome profiling by RNA next-generation sequencing; RPPA: reverse-phase protein array.

Supplementary Table 2. Comparison of direction of expression changes between the NanoString and RNASeq platforms. For this comparison, expression data from 1 non-OI bone and 1 OI type III bone were used. A total of 155 genes that fulfill NanoString quality controls were used for the validation of RNASeq differential expression data. Fold-change direction (increased or decreased) for 155 genes in OI type III bone as compared to non-OI from RNASeq and NanoString are presented. Red up-arrow depicts increased expression in OI type III bone and blue down-arrow depicts decreased expression in OI type III bone as compared to non-OI bone. Purple background: inconsistent fold change direction between the two platform; White backgrounds: consistent fold change direction between the two platforms. The consistency rate is 92%.

Supplementary Table 3. Gene Ontology (GO) analysis results with enrichment fold-change in OI type III bone. Partek GO enrichment results under the categories of skeletal processes, bone cells, collagen, and skeletal-related major signaling pathways are provided. Significance was defined by *P*-value < 0.05.

Supplementary Table 4. Top 20 gene set enrichment analysis (GSEA) results of OI type III and non-OI transcription profiles. The top 20 enriched gene set in OI type III OI or in non-OI bones are provided. NES: Normalized Enrichment Score; FDR q-val: false discovery rate adjusted q-value**.**

Supplementary Table 5. List of genes with significant change in expression in OI type III bone compared to non-OI bone. Significantly changed genes defined by |fold-change| > 2 and *P*-value < 0.05.

Supplementary Table 6. Top 20 upstream regulators predicted by IPA from RNASeq dataset and RPPA dataset in OI type III bone. The complete upstream regulator prediction results for the top 20 hits are listed together with the genes including in the input files which are downstream targets of particular individual upstream regulator. ND: the state direction (activation or inhibition) of the upstream regulator cannot be predicted.

Supplementary Table 7. Normalized RPPA dataset from 8 OI type III and 4 non-OI bones. The normalized raw intensity data of protein array for each individual sample are provided. Technical triplicates are all included. Non-OI controls include: C14, C15, C5, and C18 (columns G to O); OI type III include: OI85, OI3, OI33, OI41, OI91, OI13, OI62, and OI83 (columns P to

AO). The demographic information for these samples is provided in supplementary table 1. Protein name (AB_name), gene ID, and swiss ID are also included.

Supplementary Table 8. List of proteins with significant change in expression in OI type III bone. The complete list of significantly changed protein based on nominal P-value < 0.05 in OI type III bone as compared to non-OI bone. The protein expression level is presented as normalized intensity in the protein array.

Supplementary Table 9. Listing of all adverse events in the phase 1 clinical trial. All adverse events (AEs) reported during the trial period were recorded and were graded using the Common Terminology Criteria for Adverse Events (CTCAE). Grade 1 refers to AEs that are mild, associated with no symptoms or mild symptoms, are managed by clinical or diagnostic observations. Grade 2 refers to AEs that are of moderate severity that require minimal, local or noninvasive intervention.

Full unedited gel for Figure 2B p-SMAD2

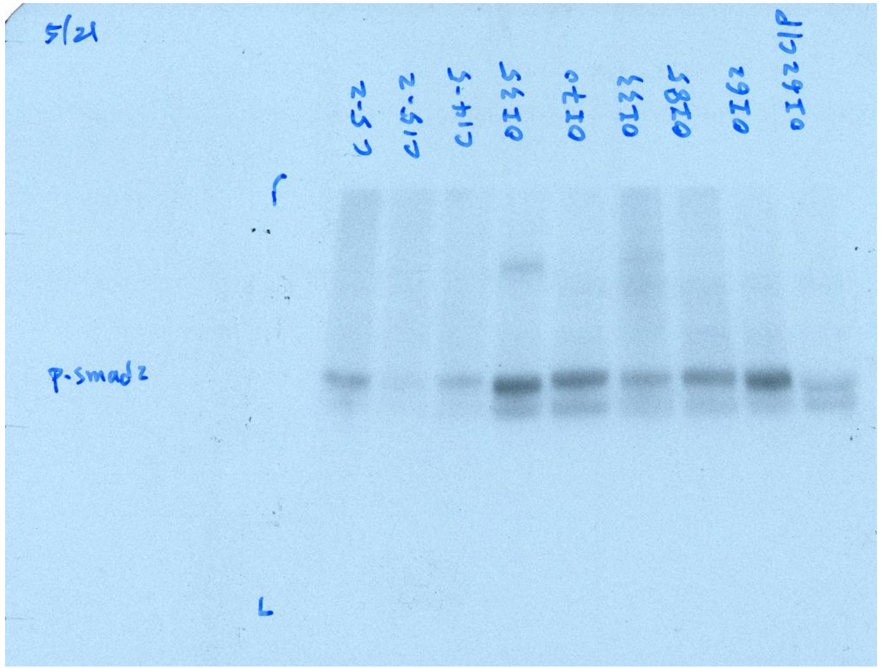

Full unedited gel for Figure 2B T-SMAD2

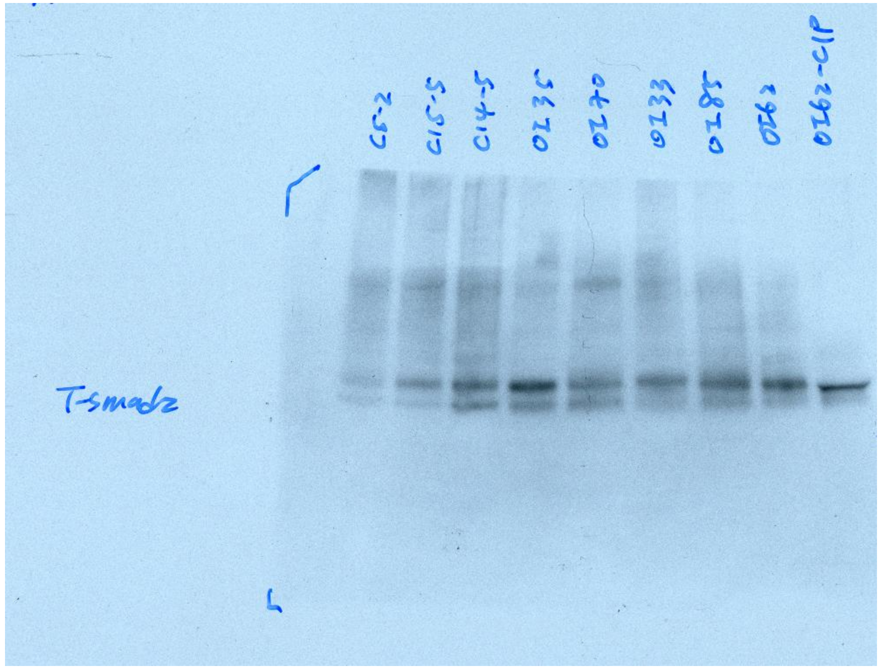

Full unedited gel for Figure 2B GAPDH

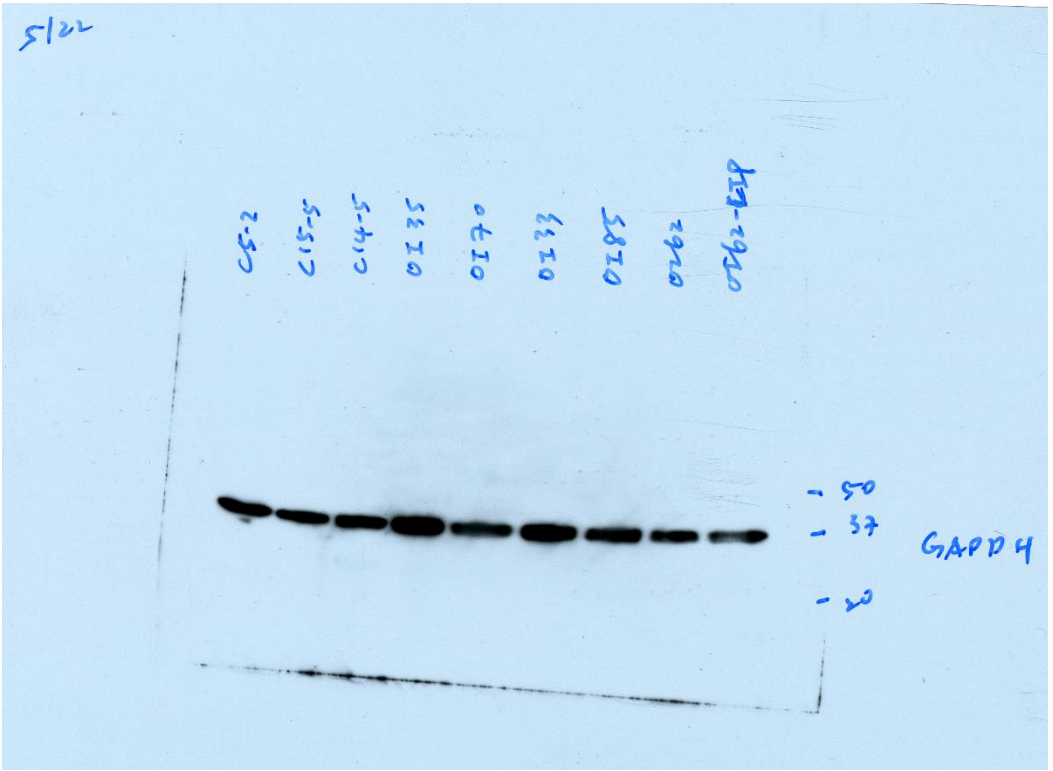

Supplement: Supplemental data [file jci-132-152571-s164.pdf]
